# Biodiversity, Anti-Trypanosomal Activity Screening, and Metabolomic Profiling of Actinomycetes Isolated from Mediterranean Sponges

**DOI:** 10.1371/journal.pone.0138528

**Published:** 2015-09-25

**Authors:** Cheng Cheng, Lynsey MacIntyre, Usama Ramadan Abdelmohsen, Hannes Horn, Paraskevi N. Polymenakou, RuAngelie Edrada-Ebel, Ute Hentschel

**Affiliations:** 1 Dept. of Botany II, Julius-von-Sachs Institute for Biological Sciences, University of Würzburg, Würzburg, Germany; 2 Strathclyde Institute of Pharmacy and Biomedical Sciences, University of Strathclyde, Glasgow, United Kingdom; 3 Hellenic Centre for Marine Research, Institute of Marine Biology, Biotechnology and Aquaculture, Gournes Pediados, Heraklion, Crete, Greece; Universidad Autonoma Metropolitana, MEXICO

## Abstract

Marine sponge–associated actinomycetes are considered as promising sources for the discovery of novel biologically active compounds. In the present study, a total of 64 actinomycetes were isolated from 12 different marine sponge species that had been collected offshore the islands of Milos and Crete, Greece, eastern Mediterranean. The isolates were affiliated to 23 genera representing 8 different suborders based on nearly full length 16S rRNA gene sequencing. Four putatively novel species belonging to genera *Geodermatophilus*, *Microlunatus*, *Rhodococcus* and *Actinomycetospora* were identified based on a 16S rRNA gene sequence similarity of < 98.5% to currently described strains. Eight actinomycete isolates showed bioactivities against *Trypanosma brucei brucei* TC221 with half maximal inhibitory concentration (IC_50_) values <20 μg/mL. Thirty four isolates from the Milos collection and 12 isolates from the Crete collection were subjected to metabolomic analysis using high resolution LC-MS and NMR for dereplication purposes. Two isolates belonging to the genera *Streptomyces* (SBT348) and *Micromonospora* (SBT687) were prioritized based on their distinct chemistry profiles as well as their anti-trypanosomal activities. These findings demonstrated the feasibility and efficacy of utilizing metabolomics tools to prioritize chemically unique strains from microorganism collections and further highlight sponges as rich source for novel and bioactive actinomycetes.

## Introduction

Marine sponges are known to maintain dense and diverse microbial communities [1]. Current hypothesis holds that their microbial symbionts may at least in some cases, contribute to the sponges’ protection by producing chemical substances as defense against sponge predators and sponge diseases [2]. Indeed, a growing number of bioactive secondary metabolites have been isolated from marine sponge-associated bacteria which provides indirect support to this hypothesis, but more notably feeds the marine drug discovery pipeline [3–8]. The marine sponge-associated bacteria within the order Actinomycetales (class Actinobacteria), and herafter colloquially termed “actinomycetes”, have proven to be a particularly prolific source of bioactive natural compounds [9–17]. Many novel species have been isolated from marine sponges [18–21] and the biological novelty also affords structurally new, bioactive compounds [11,12,15,22].Efforts are on-going to discover novel actinomycete diversity from sponges and other marine invertebrates with the overarching aim to explore marine-derived compounds for drug discovery.

In microbial drug discovery programs, compound isolation from large strain collections is frequently labor-intensive and time-consuming. To reduce the rediscovery of known compounds, dereplication of the microbial isolates prior to further chemical work is one useful measure. Traditionally, the selection of candidate strains from strain collections depended on bioactivity screening [9]. However, bioactivity data alone does not provide information on the underlying chemical entities. Similarly, 16S rRNA gene sequence based phylogenetic data alone are not sufficient as related strains do not necessarily produce the same secondary metabolites and strains belonging to different genera may be chemically similar [23]. Consequently, a strategy using chemical dereplication coupled with multivariate analyses was recently established and is frequently employed in microbial drug discovery programs [24–27]. In this strategy, NMR and LC-MS based analytical techniques are utilized to initially assess and dereplicate secondary metabolites of microbial extracts. Multivariate analysis such as principal component analysis (PCA) and orthogonal partial least squares—discriminant analysis (OPLS-DA) analysis are then performed using metabolomics data to identify the chemically distinct strains that may yield novel bioactive secondary metabolites. From a chemical perspective, this approach covers the inherent shortages of bioactivity- and taxonomy-based dereplication and provides for an efficient pipeline in the screening of microbial strain collection.

In the present study, actinomycetes were cultivated from various eastern Mediterranean sponge species and phylogenetically characterized based on nearly complete 16S rRNA gene sequencing. The actinomycete isolates were further subjected to anti-trypanosomal bioassays and metabolomics analysis. The obtained data were integrated to prioritize selected actinomycetes for follow-up chemical isolation and structure identification work.

## Materials and Methods

### Specimen collection

Sponge samples were collected from the islands of Milos and Crete, Greece, located in the eastern Mediterranean Sea. The eastern basin of the Mediterranean Sea is considered to be one of the most oligotrophic regions in the world with relative warm (~15–25°C) and high saline waters (36–40 psu) [28]. The microbial diversity of sediment samples from this geographic location had been previously explored and actinobacteria were found to be a dominant community member in at least one sample [29]. The sponges *Agelas oroides*, *Chondrilla nucula*, *Chondrosia reniformis*, *Ircinia variabilis*, *Petrosia ficiformis*, *Spirastrella cunctatrix*, *Sarcotragus foetidus*, and *Sarcotragus spinosulus* were collected by SCUBA diving at 5–7 m depth from offshore Pollonia, Milos, Greece (N36.76612°; E24.51530°) in May 2013. The island of Milos lies in the centre of the Hellenic volcanic arc in the convergence zone between the African and Aegean tectonic plates. Most parts of the island are covered by volcanic rocks and hydrothermal vents [30]; however hot emissions were not observed at the sampling site. The sponge species *Axinella damicornis*, *Axinella cannabina*, *Agelas oroides*, *Aplysina* sp., *Acanthella acuta*, *Chondrosia reniformis*, *Chondrilla nucula*, *Dysidea avara*, *Ircinia fasciculata*, *Petrosia ficiformis*, *Phorbas tenacior*, and *Sarcotragus* sp. were collected by SCUBA diving at 3–28 m depth from offshore Agios Ioannis, Souda Bay, Crete, Greece (35.47032° N and 24.12508° E) in Nov 2013. Souda Bay is an area of intense human activities and a strategic naval base where shipping, coastal fisheries, aquaculture, tourism and other activities occur. In both sampling events, diving was performed by sponge taxonomy experts from HCMR. Sponge species were first identified based on sponge morphology and subsequently by external and internal morphological characteristics (i.e, spiculae).

### Ethics Statement

The present study involved collection of sponge samples. Sampling did not involve endangered or protected species, although they may have occurred at or near the sites sampled. Sampling was performed by HCMR which is a national body under the auspices of the Hellenic Ministry of Culture, Education and Religious Affairs. Permissions to access Milos and Crete sampling sites by SCUBA diving were obtained prior to onset of this study by local port authorities (port authorities of Milos and Souda, respectively). HCMR may be contacted for future permissions.

### Bacterial cultivation

Sponges were rinsed with sterilised seawater which had been filtered by use of a 0.2 μm filter pump to remove seawater bacteria. One cm^3^ of sponge inner tissue was excised and homogenized in 10 mL seawater using mortar and pestle to access the sponge symbiont consortia residing internally within sponge tissues. After 10 min to allow for the settlement of particulate material, the supernatant was taken in duplicate and diluted with seawater in ten-fold series (10^−1^, 10^−2^, 10^−3^). The supernatants were heated in the heating block at 90°C for 10 min to kill the fast growing bacteria and to enrich for spore-forming actinomycetes. One hundred μl of heated and non-heated supernatants were used for inoculation per each agar plate.

Twelve different agar media were used including the basic actinobacterial media M1 [31], ISP medium 2 [32], and Oligotrophic medium (OLIGO) [33]. All media were prepared either in arteficial or natural seawater. The media M1_SE and OLIGO_SE, were further supplemented with 1% corresponding sponge biomass extract (1 g/mL) [21], as indicated by the abbreviation “_SE”. Nutritionally poor media were generated by adding only artificial seawater (ASW) or natural seawater (NSW) to agar. Attempts to enrich for filamentous actinomycetes were undertaken on M1_F and ISP medium 2_F which were covered by soft agar (as indicated by the abbreviation “_F”). The media R2A [34], MA medium [34], and M5 [35] were additionally used to maximize actinomycete diversity. All media were supplemented with the antifungal agent cycloheximide (0.2 μm pore size filtered; 100 μg/mL) and nalidixic acid (25 μg/mL) which inhibits many fast growing Gram-negative bacteria [21]. One hundred μL of bacterial homogenate supernatants was plated out on agar plates in duplicate respectively using sterile glass beads. The plates were incubated at 30°C and inspected regularly for growth for up to 6 weeks. Actinomycete-like colonies (leathery texture, dry or folded appearance, aerial and/or substrate mycelium, presence of diffusible pigments) were re-streaked several times on corresponding agar media until colonies were visually free of contamination. The isolated actinomycetes were stored in 87% glycerol in ISP2 and maintained in cryotubes at—80°C.

### Phylogenetic analysis

Genomic DNA was extracted using the FastDNA spin kit for soil (MP Biomedicals, Germany) following manufacturer’s instructions. 16S rRNA gene amplification and sequencing were performed using the universal primers 27F (5ʹ-GAGTTTGATCCTGGCT CAG-3ʹ) and 1492R (5ʹ-GGTTACCTTGTTACGACTT-3ʹ) as described previously [36]. Sequences were checked for chimeras using the DECIPHER web service [37]. The remaining sequences were quality-filtered from both ends with the BWA's trimming algorithm and a quality threshold of 30 [38]. To obtain consensus sequences, a self-written perl script calculating a MUSCLE alignment and considering quality scores on overlap positions was performed [39]. Alignments were manually curated if necessary. Nearest related and type strain sequences were searched through a BLAST run against the non-redundant and 16S ribosomal database [40]. The phylogenetic tree was based on a multiple alignment generated with the SINA web aligner [41]. A maximum likelihood tree with 500 bootstrap repetitions was constructed by means of RaxML [42] and finally visualized via the iTOL web service [43].

### Extracts preparation and bioactivity assays

The strains were fermented in 250 mL ISP medium 2 broth in 500 mL conical flasks with shaking (shaker, Edmund Bühler, Germany) at 175 rpm at 30°C in the incubator (Binder, Germany) and harvested based on their individual growth pattern after 5–10 days. Only one medium was used for fermentation in order to ensure comparability between the different strains in metabolomics analyses. The filtrate of the fermented culture was extracted twice with 250 mL ethyl acetate for each time. The extracts were generated by evaporating the solvent and 0.2 mg of each extract was used for anti-trypanosomal activity. ISP2 broth medium control was performed to ensure the purity and production of the fermentations. The anti-trypanosomal bioassay was performed following Huber and Koella [44]. Briefly, 10^4^ trypanosomes per mL of *Trypanosoma brucei brucei* strain TC 221 were cultivated in Complete Baltz Medium. Trypanosomes were tested in 96-well plate chambers against different concentrations of test extracts at 10–200 μg/mL in 1% DMSO to a final volume of 200 μL. For controls, 1% DMSO as well as parasites without any test extracts were used simultaneously in each plate to show no effect of 1% DMSO. The plates were then incubated at 37°C in an atmosphere of 5% CO_2_ for 24 h. After addition of 20 μL of Alamar Blue, the activity was measured after 48 and 72 h by light absorption using an MR 700 Microplate Reader (Dynatech Engineering Ltd., Willenhall, UK) at a wavelength of 550 nm with a reference wavelength of 650 nm. The IC_50_ values of extracts were quantified by linear interpolation in three independent measurements.

### Metabolomics analysis

LC-HRMS experiments were carried out on an Accela HPLC from Thermo Scientific (Bremen, Germany) combined with Accela UV/VIS and Exactive (Orbitrap) mass spectrometer from Thermo Fisher Scientific (Bremen, Germany). The subjected extracts were prepared in MeOH at a concentration of 1 mg/mL. A HiChrom, ACE (Berkshire, UK) C18, 75 mm × 3.0 mm, 5 μm column was attached to the HPLC. The mobile phase consisted of purified water (A) and acetonitrile (B) with 0.1% formic acid in each solvent. The gradient started with 10% B and was linearly increased to 100% B at a flow rate of 300 μL/min within 30 min and remained isocratic for the next 5 min before linearly decreasing back to 10% B for the following 1 min. The mobile phase was then equilibrated for 9 min before the next injection and the total analysis time for each sample was 45 min. The injection volume was 10 μL, and the tray temperature was maintained at 4°C and the column oven at 20°C. High resolution mass spectrometry was carried out in both positive and negative ionisation modes with a spray voltage at 4.5 kV and capillary temperature at 320°C. The mass range was set from m/z 100–2000 for ESI-MS (Electrospray Mass Spectrometry) using in-source CID (Collision-Induced Dissociation) mechanism and m/z 50–1000 for MS/MS using untargeted HCD (High Energy Collision Dissociation).

Data-dependent MS^2^ and MS^3^ experiments were performed using a Finnigan LTQ Orbitrap coupled to a Surveyor Plus HPLC pump (Thermo Scientific, Bremen, Germany) and autosampler (Thermo Fisher, Bremen, Germany) in positive and negative ionization modes using a mass range of m/z 100–2000 and 30,000 resolution. The capillary temperature was 270°C, the ion spray voltage was 4.5 kV, the capillary voltage 35 V, the tube lens voltage 110 V and the sheath and auxiliary gas flow rates were 50 and 15, respectively (units not specified by manufacturer). Multi-fragmentation (MS^n^) experiments were accomplished on an Orbitrap analyzer, CID (collision-induced dissociation) was utilized with a normalized collision energy of 35%, activation Q of 0.250 ms and activation time of 30,000 ms applied on ions of most intense, 2nd most intense, and 3rd most intense peaks for MS^2^ and MS^3^, respectively, at an isolation width of 3 microns with 5 microscans. Resolution was at 15,000 m/Δm50%, while the minimum ion signal threshold was set to 500. Fragment mass tolerance for molecular formula detection was set at ±5 ppm [24].

Raw LC-MS data were initially sliced by MassConvert tool ProteoWizard [45] into positive or negative files in mzML format. The sliced data sets were imported into MzMine 2.12, a framework for differential analysis of mass spectrometry data. Peak detection in MZmine 2.12 was executed following noise removal, chromatogram construction, and peak deconvolution. First, the mass values were detected using the centroid mode in each spectrum and the peaks below 1×10^4^ of the height were discarded as noise. In the second step, chromatograms were constructed for each of the mass values which span over a certain time range. The minimum time span over the same ion was set as 0.2 min and the error of the ion *m/z* value was allowed within 5 ppm. The minimum intensity of the highest data point in the chromatogram was set at 1×10^4^. Finally, a deconvolution algorithm was applied to each constructed chromatogram of each mass ion to recognise the actual chromatographic peaks. The “local minimum search” algorithm which searches for local minima in the chromatogram and separates individual peaks at minimal points was used. The settings to separate individual peaks were as follows: the chromatographic threshold at 95%; search minimum in RT range of 0.4 min; minimum relative height at 5%; minimum absolute height of 3×10^4^; minimum ratio of peak top/edge 3 and peak duration range from 0.2 to 5.0 min. The seperated peaks were then deisotoped using the function of isotopic peaks grouper in which we set m/z tolerance at 0.001 m/z or 5.0 ppm; retention time tolerance at 0.1 absolute (min); maximum charge of 2; and representative isotope being most intense. Retention time normalizer was also used after deisotoping to reduce inter-batch variation by setting m/z tolerance at 0.001 m/z or 5.0 ppm; retention time tolerance at 0.5 absolute (min), and minimum standard intensity: 5.0 × 10^3^. The remaining peaks in different samples were aligned based on the mass and retention time of each peak. The ion *m/z* tolerance for alignment was set at 5 ppm, retention time was 5 relative (%), and weight for *m/z* and Rt were 20 respectively. Following alignment, the resulting peak list was gap-filled with missing peaks using the intensity tolerance of 25% and retention time tolerance of 0.5 min. The solvent peaks were subtracted from samples by peak intensity at a level above 1 × 10^5^. The medium effects were then cleaned up by using an Excel program which was written to subtract of medium peaks but remain features which are 20 times greater in the samples than in the medium. Data were then imported to SIMCA for further multivariate analysis [24].

NMR measurements were carried out on Fourier Transform NMR spectrometer JNM LA-400 400 MHz instruments (JEOL, Japan). All extracts were prepared in DMSO-d6 at a concentration of 10 mg/mL. The preprocessed ^1^H NMR spectra including the medium blank were stacked into one plot, binned using average sum and integral region of 0.01 ppm, and normalised to the intensity by the largest peak (value 100) in Mnova (Mestrelab Research SL, US). The binned spectral data between δ 0.5 ppm to δ 12.5 ppm were exported to Excel in ASCII format followed by medium effect removal and solvent peak removal (DMSO-d6 at δ 2.47–2.51 ppm). The resulting peak list was imported into SIMCA 13.0.3 for further multivariate analysis.

## Results and Discussion

### Strain isolation and phylogenetic identification

A total of 64 actinomycete isolates were obtained from the Greek collection effort. The isolates were phylogenetically affiliated to 23 genera, 15 families and 8 suborders based on nearly complete 16S rRNA genes sequencing (each> 1100 bp in length, S1 Table). The sponge *Sarcotragus spinosulus* yielded the highest number of actinomycetes (12), followed by *Petrosia ficiformis* (10), *Spirastrella cunctatrix* (10), *Agelas oroides* (9), *Phorbas tenacior* (5), *Chondrilla nucula* (4), *Ircinia variabilis* (4), *Ircinia fasciculata* (3), *Sarcotragus foetidus* (3), *Axinella damicornis* (2), *Acanthella acuta* (1), and *Aplysina* sp. (1), (S1 Fig). *Petrosia ficiformis* was notable for the high recovery rate of *Streptomyces* isolates (n = 5). No actinomycetes were isolated from *Chondrosia reniformis*, *Dysidea avara*, *Sarcotragus* sp., and *Axinella cannabina*. Even though the same procedure was applied to each sponge species, the yields are not quantitatively comparable, because bacterial colonies were picked based on colony diversity rather than on colony abundance.

**Fig 1:**
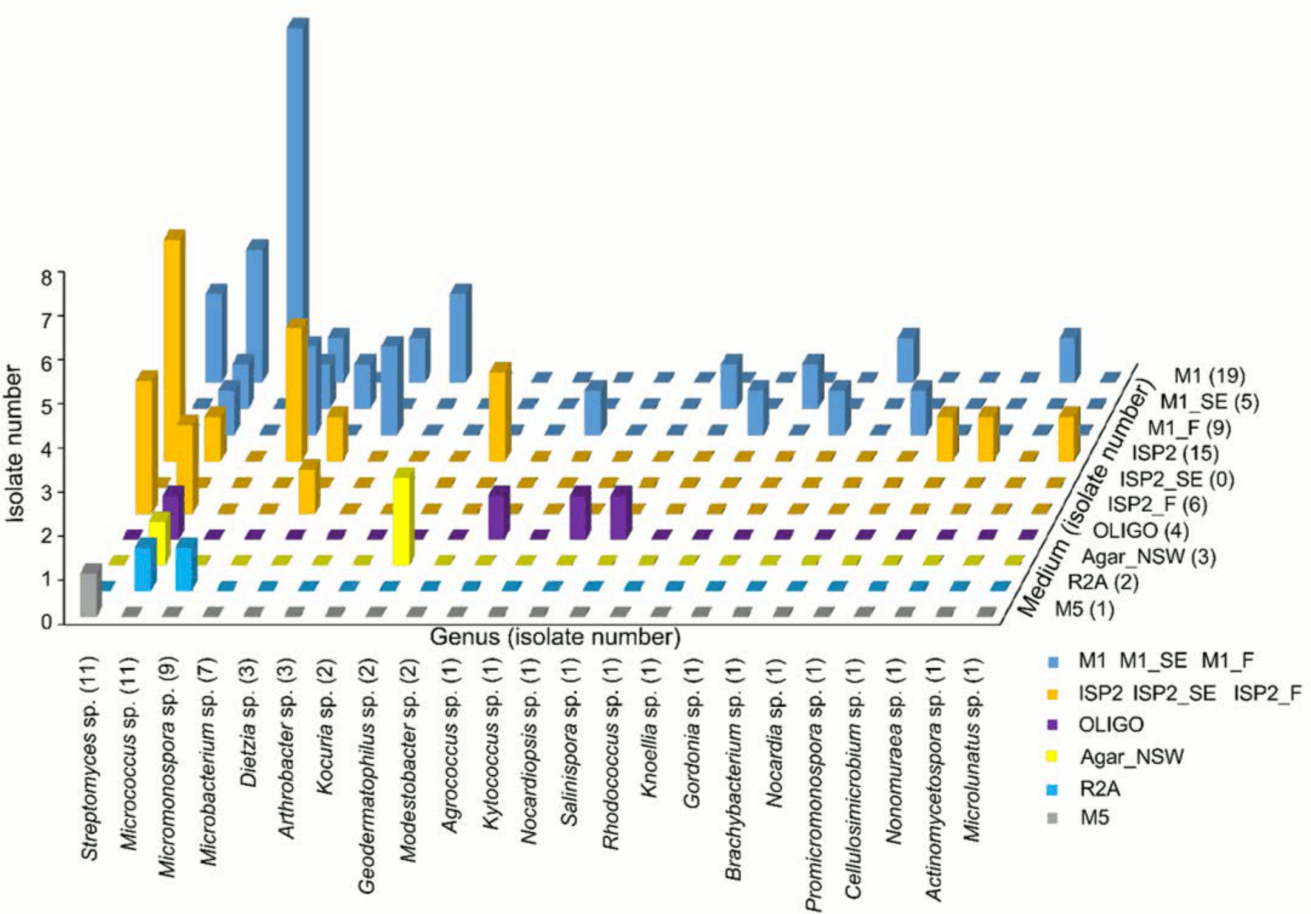
Distribution of actinomycete genera grown on different media compositions.

Among the 64 actinomycete isolates, the genera *Streptomyces* (n = 11), *Micrococcus* (n = 11), and *Micromonospora* (n = 9) were numerically dominant which is consistent with previous studies [21], followed by *Microbacterium* (n = 7), *Dietzia* (n = 3), *Arthrobacter* (n = 3), *Kocuria* (n = 2), *Geodermatophilus* (2), *Modestobacter* (2), as well as an additional 14 genera that were represented by only one isolate (Fig 1). Representatives from genera *Geodermatophilus*, *Microlunatus*, *Actinomycetospora*, *Modestobacter*, and *Promicromonospora* were, to our knowledge, isolated from marine sponges for the first time. With respect to medium composition, M1 yielded the highest recovery rate (51.6%) with 33 isolates representing 15 different genera and showed further the best recovery rates of *Micromonospora* (8 isolates). The ISP2 medium exhibited the second best recovery rates (32.8%) with a total of 21 isolates and representing a total of eight genera. Among them, eight isolates were assigned to *Streptomyces*. One putatively new isolate, *Rhodococcus* sp. SBT367, was recovered from M1_SE medium which was amended with sponge extract. Other amended media (identified by the label “_F”), prepared to enrich for filamentous *Streptomyces* sp., resulted in the isolation of three *Streptomyces* sp. (SBT344, SBT345, and SBT348). The media OLIGO and Agar_NSW yielded 4 and 3 actinomycetes, respectively, and R2A and M5 yielded one actinomycete species each.

**Fig 2:**
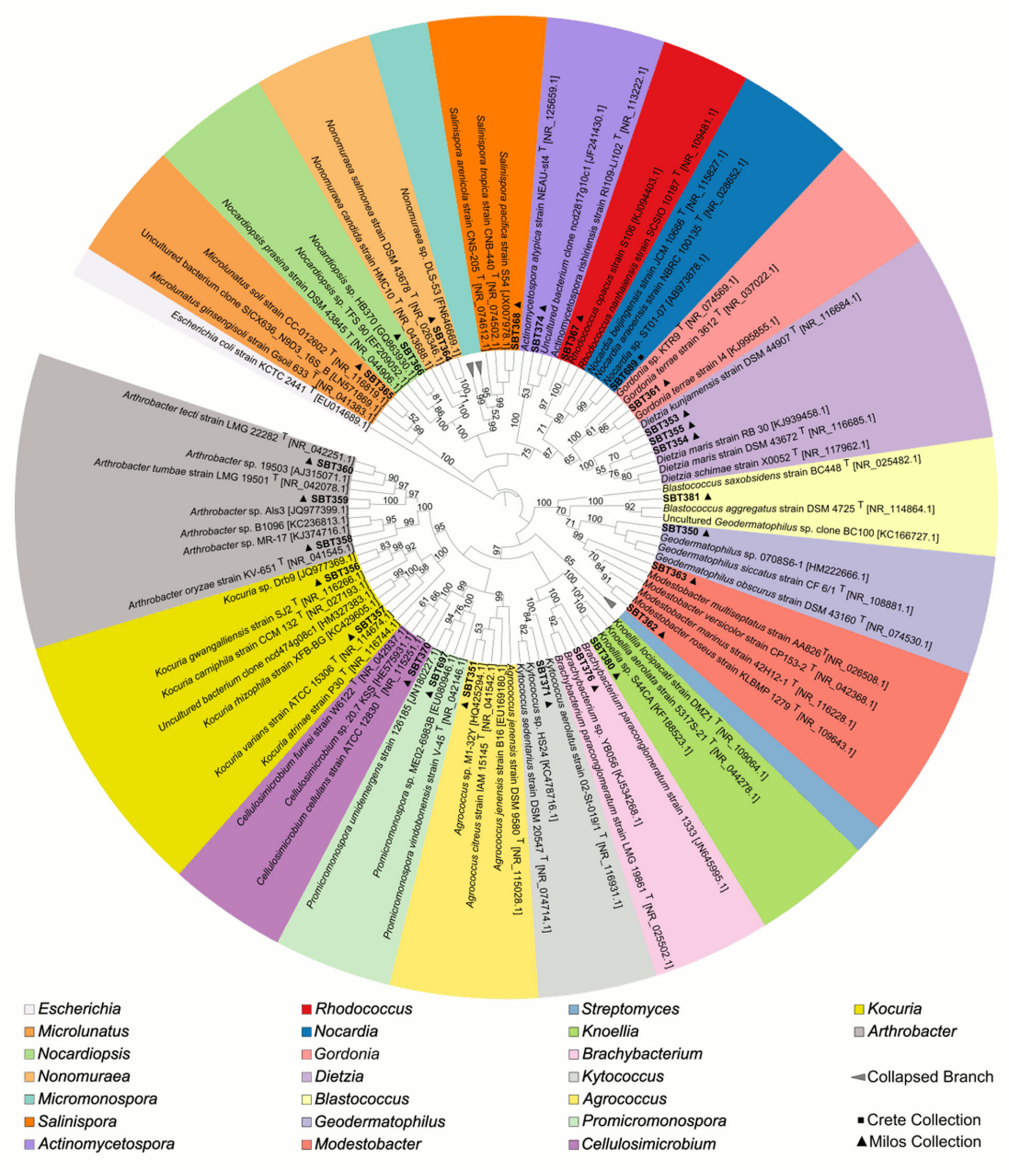
Maximum-likelihood tree of selected actinomycete isolates from the Crete (SBT 686–697) and Milos collection (SBT349-381) as well as their nearest representative strains based on the 16S rRNA gene sequence. The color legend indicates the genus-level of all sequences. The tree is rooted at *Escherichia coli* strain KCTC 2441^T^ which serves as outgroup. All bootstrap values >50 (500 resamples) are given in percent at the nodes of the tree.

A total of 46 isolates were selected from the strain collection based on their unique colony morphology. Secondly, isolates from genera known to be prolific producers of secondary metabolites were prioritized. A maximum-likelihood tree was constructed and the closest type strains as well as the closest strains found in the NCBI nt database for each of the isolates were included (Fig 2). The two major taxonomic groups from this study (i.e. *Micromonospora* and *Streptomyces*) were collapsed (Fig 2) and shown at higher resolution (S2A and S2B Fig). All genera fell into distinct clades, with the exception of the genus *Salinispora*, which fell within the *Micromonospora*; albeit with low bootstrap support of 52 (S2A Fig). This is unsurprising since *Salinispora* and *Micromonospora* are closely related. The isolates SBT354 and SBT355 formed a distinct clade within the genus *Dietzia* and they may be the same strain since they were derived from the same *Sarcotragus spinosulus* sample (Fig 2). The isolates SBT345 and SBT690, from *Agelas oroides* and *Ircinia fasciulata* respectively, formed a separate cluster within the genus *Streptomyces*. These isolates may belong to the same species besides the fact that they were obtained from different sponge species and sample sites (S2B Fig). In terms of novelty, four bacteria exhibited 16S rRNA gene sequence similarities < 98.5% compared to 16S rRNA gene sequences from other isolates available in the NCBI database. These were affiliated with the genera *Geodermatophilus* (SBT350), *Microlunatus* (SBT365), *Rhodococcus* (SBT367), and *Actinomycetospora* (SBT374) (S1 Table). The 16S rRNA gene sequences of all isolates were deposited in GenBank under the accession numbers KP145919-KP145922 and KP238412-KP238453.

### Anti-trypanosomal assay

The anti-trypanosomal assay is used here as one way to discriminate between the active and inactive extracts to assist the multivariate statistical analysis. All actinomycete extracts except those belonging to the chemically poor genera *Micrococcus* and *Microbacterium* were tested in the anti-trypanosomal assay. Of the 46 tested extracts, eight (8) were active against *T*. *brucei brucei* strain TC 221 with IC_50_ values < 20 μg/ml (Table 1). Among them, extracts of three strains of *Streptomyces* sp. SBT344, *Nonomurea* sp. SBT364, and *Modestobacter* sp. SBT363 exhibited potent inhibition with IC_50_ values <10 μg/ml after 48 h and 72h incubation. To our knowledge, bioactivities are reported here for the first time for the genera *Modestobacter* and *Geodermatophilus*.

**Table 1:** Anti-trypanosomal activities of sponge associated actinomycetes.

Isolate	IC_50_ (48h) μg/ml	IC_50_ (72h) μg/ml
***Streptomyces* sp. SBT344**	<10	<10
***Streptomyces* sp. SBT348**	16.52	20.50
***Modestobacter* sp. SBT362**	19.34	21.28
***Modestobacter* sp. SBT363**	<10	<10
***Nonomuraea* sp. SBT364**	<10	<10
***Rhodococcus* sp. SBT367**	19.97	22.37
***Geodermatophilus* sp. SBT381**	18.60	21.36
***Micromonospora* sp. SBT687**	14.87	19.95

### Strain prioritization using metabolomics

#### Milos Collection

**Fig 3:**
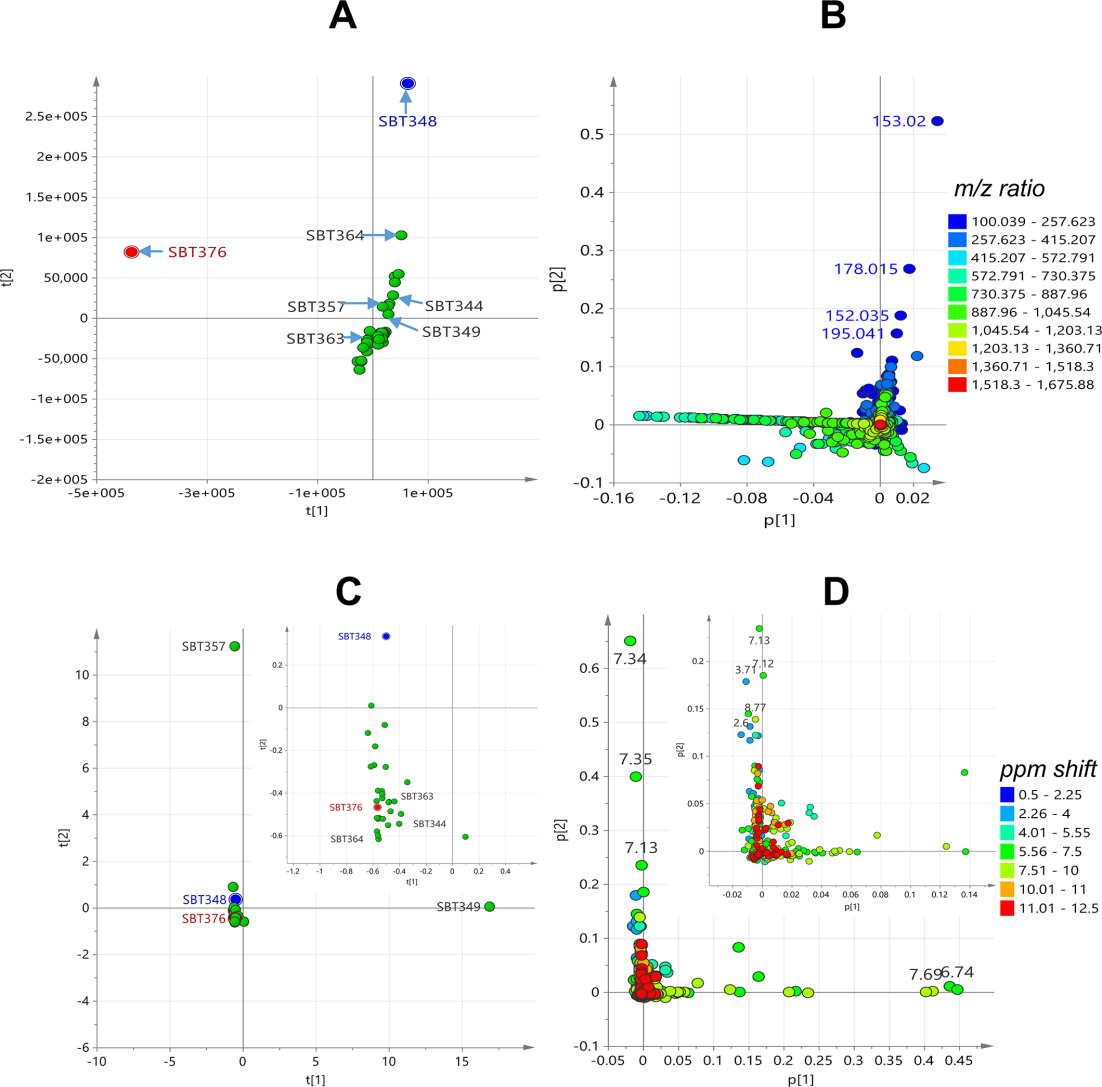
PCA analysis of 34 extracts from Milos collection. Scores plot (A) and loadings plot (B) of LC-HRMS data; scores plot (C) and loadings plot (D) of ^1^H NMR data of 34 extracts. For the scores plot (A and C), the outmost PCA outliers for the HRMS data were colored blue for the antitrypanosomal active extract and red for the inactive extract. Inset shows expansion of the central field of the loadings plot to reveal the dense distribution of resonances between 10.0 and 12.5 ppm.

**Fig 4:**
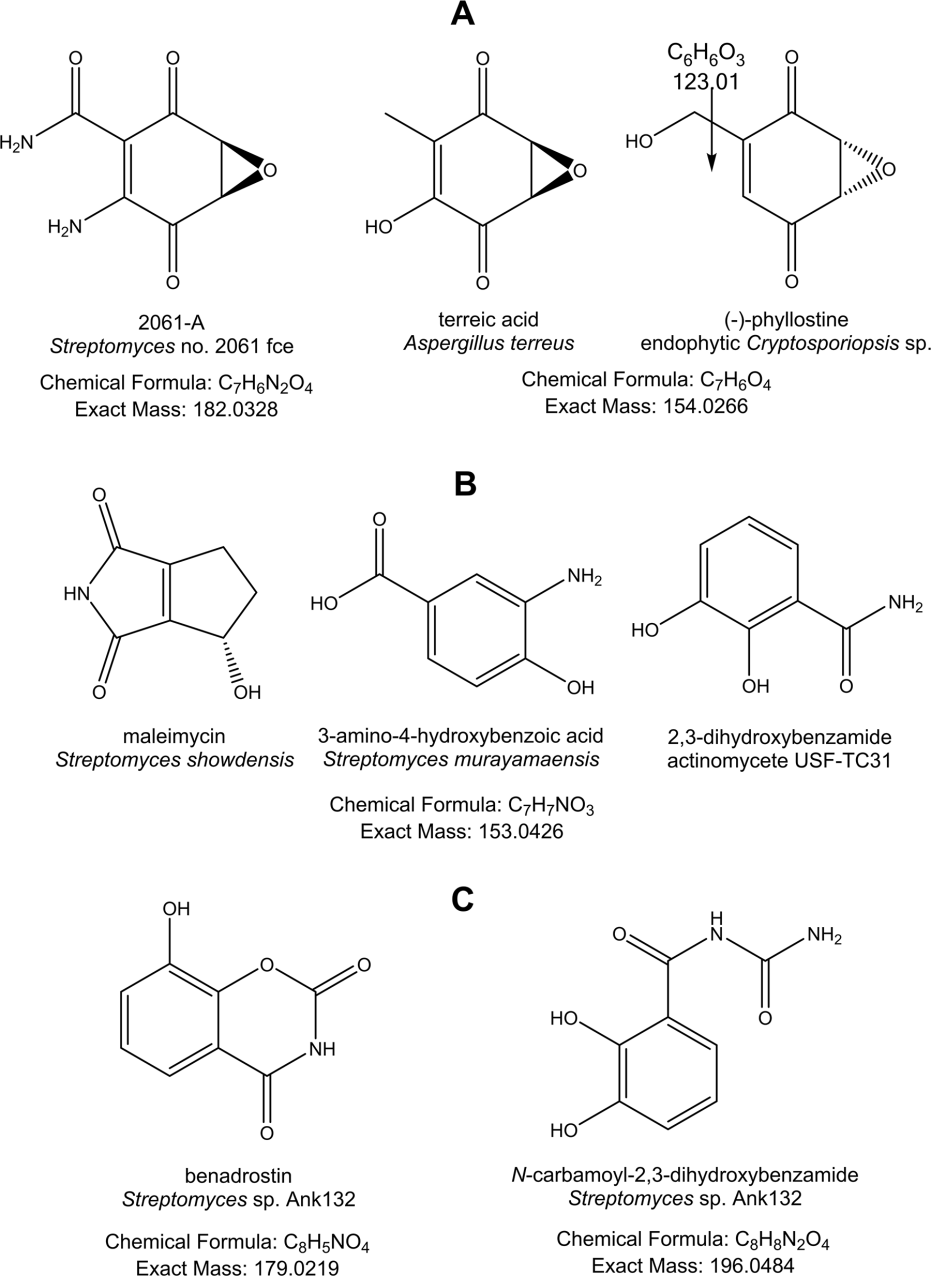
Dereplicated metabolites for the outlying mass ion peaks for *Streptomyces* sp. SBT348. (A) Compound “hits” for *m/z* 153.020 [M-H]^-^ and structurally related metabolites from genus *Streptomycete*, (B) compound “hits” for *m/z* 152.035 [M-H]^-^, and (C) *m/z* 178.015 [M-H]^-^, and 195.041 [M-H]^-^. Stereochemistry is shown as predicted from the MarinLit database.

PCA analysis was performed on the LC-MS data of 34 isolates and generated from four components, a R2 (coefficient of determination) value at 0.524 was achieved using the Pareto scaling mode, indicating a good mode for the data to fit (R2 > 0.5). The two outliers, *Streptomyces* sp. SBT348 and *Brachybacterium* sp. SBT376, were revealed in the scores plot (Fig 3A), thus indicating their chemical uniqueness. The loadings plot (Fig 3B) represented the molecular ion masses of the detected secondary metabolites. Those at the same quadrant position as the outliers were the significant secondary metabolites that contribute to the specific observations in the scores plot (Fig 3A). From the loadings plot, the outlying features of SBT348 were caused by low molecular weight metabolites from 100 to 250 Da while SBT376 yielded metabolites with molecular weights between 550 and 720 Da (Fig 3B). By comparing the results with the bioassay screening, only SBT348 showed bioactivity against *T*. *brucei brucei* strain TC 221 (Table 1). The outlying mass ion peak at *m/z* 153.020 [M−H]^−^ for the predicted molecular formula C_7_H_6_O_4_ was putatively identified as either terreic acid [46–49] or (−)-phyllostine [50,51], both earlier described as fungal metabolites. The oxabicyclo-hept-3-ene-2,5-dione structure found in terreic acid and phyllostine is structurally related to 2061-A (G7063-2) previously isolated from a *Streptomyces* species [52,53] (Fig 4A). MS^2^ data obtained by source fragmentation gave an ion peak at *m/z* 123.008 [M-CH_3_O]^-^ for C_6_H_6_O_3_ which indicated the presence of a similar substituent found in (−)-phyllostine (Fig 4A). Outlying mass ion peak at *m/z* 152.035 [M−H]^−^, for the predicted molecular formula C_7_H_7_NO_3_, was dereplicated as maleimycin [54,55], 3-amino-4-hydroxybenzoic acid [56], or 2,3-dihydroxybenzamide [57], all of which were previously reported as actinomycete metabolites (Fig 4B). While outlying mass ion peaks at *m/z* 178.015, and 195.041, both [M−H]^−^, were tentatively identified as benadrostin [58,59] and N-carbamoyl-2,3-dihydroxybenzamide [57], respectively, both isolated from *Streptomyces* sp. Ank132 (Fig 4C). Due to low abundance of the ion peak intensity at *m/z* 152.035, 178.015 and 195.041, it was not possible to obtain any MS^2^ data by source fragmentation. To validate the dereplication data for the low abundance ion peaks, SBT348 was subjected to higher-energy collisional dissociation on the Orbitrap. As illustrated in Fig 4B and 4C, respectively, the loss of an amide group was corroborated to *m/z* 152.035 for 2,3-dihydroxybenzamide as indicated by the fragment ion at *m/z* 109.03 [C_6_H_5_O_2_] and *m/z* 178.015 for benadrostin that gave a fragment ion at *m/z* 137.02 [C_7_H_5_O_3_]. For *m/z* 195.041, the dereplicated metabolite did not match the MS^2^ and MS^3^ data as shown at *m/z* 166.05 [−NO] and 151.04 [−NH_2_] which indicated the occurrence of an amidoxime functional group instead of a *N*-carbamoyl-amide moiety (Fig 4C). LC-MS/MS analysis of *Streptomyces* sp. SBT348 extract gave significant MS^2^ and MS^3^ data for molecular ion peaks between 200 and 600 Da (S2 Table), which interestingly did not get any matching hit from the dereplication study. It can be observed that the predicted molecular formula from the high resolution data were highly oxygenated indicating a very good lead for biological activity of the projected secondary metabolites produced by *Streptomyces* sp. SBT348.

Proton NMR data of thirty four strains from the Milos collection were also subjected to PCA analysis (Fig 3C), which was generated from a two component model achieving a R2 value at 0.719 using the Pareto scaling mode. Two outlier strains, *Streptomyces* sp. SBT349 and *Kocuria* sp. SBT357, were revealed distant from the main cluster (Fig 3C). The significant chemical shifts contributing to the isolation of the outliers were revealed in the loadings plot matching the same quadrant position in the scores plot (Fig 3D). For SBT349, the outlying chemical shifts were found in the aromatic region. The ABCD aromatic system was indicated by the 8 Hz coupling constant and signal correlation between a doublet-triplet-triplet-doublet resonances as revealed in the ^1^H and COSY spectra (S3 Fig). Its HMBC data confirmed that the major compound was likely anthranilic acid [60–63] (S3 Fig). The presence of anthranilic acid as a major component was also shown in the dereplication of the LC-HRMS data which indicated a peak at 3.47 min and *m/z* 138.0551 [M+H]^+^ establishing the molecular formula C_7_H_8_NO_2_. For SBT357, the unique chemical shifts at δ_H_ 7.34 and 7.35 also suggested the presence of an aromatic structure which is usually chemically interesting. However, using this statistical model, correlation between the outliers and antitrypanosomal bioactivity was not observed. Both SBT349 and SBT357 were found inactive against *T*. *brucei brucei* strain TC 221. The observed outliers, SBT348 and SBT376, from the PCA analysis of HRMS data were both clustered at the center of the scores plot (Fig 3C). Expansion of the central field of the loadings plot (Fig 3D) revealed a dense distribution of resonances between 10.0 and 12.5 ppm indicating the occurrence of highly deshielded exchangeable protons found in phenolic O*H* substituents, aromatic/olefinic-bound N*H*_*2*_s and amide moieties. This validates the dereplication result of the HRMS data indicating the presence of such structures (Fig 4) described above. The COSY spectrum (S4 Fig) also displayed cross peaks between resonances at 6.50 to 7.50 ppm which are the expected correlations that would be observed for 3-amino-4-hydroxybenzoic acid, 2,3-dihydroxybenzamide, benadrostin, and N-carbamoyl-2,3-dihydroxybenzamide (Fig 4).

**Fig 5:**
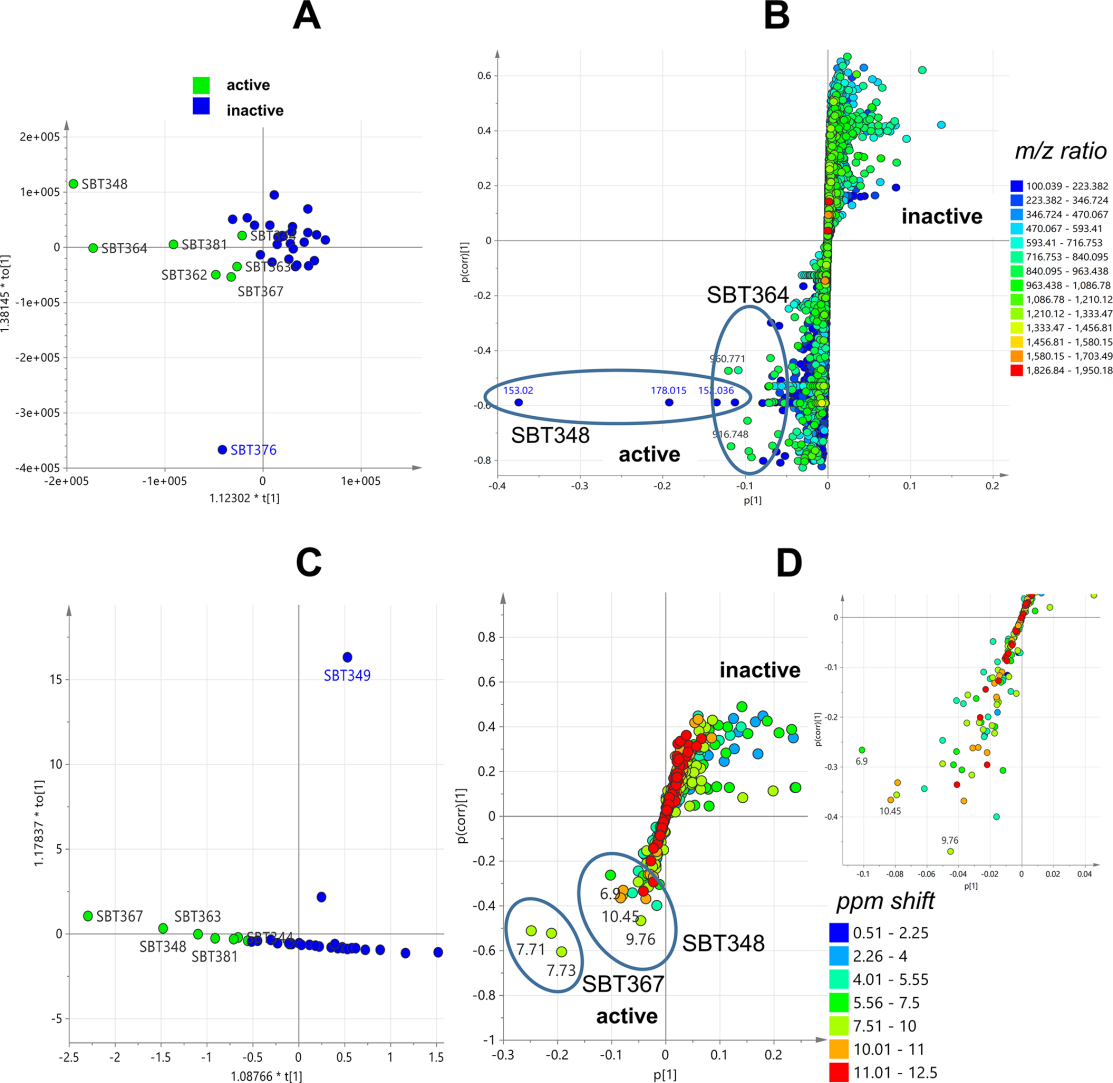
OPLS-DA analysis of 34 extracts from Milos collection. Scores plot (A) and S plot (B) of LC-HRMS data; scores plot (C) and S plot (D) of ^1^H NMR data of 34 extracts. Inset shows expansion of the lower left quadrant representing those of the active group.

To be able to predict and understand the type of chemistry that would be responsible for the bioactivity of the seven (7) extracts obtained from the Milos sponges (Table 1), a supervised multivariate analysis was accomplished by subjecting both the HRMS (Fig 5C and 5D) and ^1^H NMR (Fig 5C and 5D) data sets to OPLS-DA (Orthogonal Partial least squares-Discriminant Analysis). The sample extracts were divided in to two classes: active vs. inactive (Y variables) and by using an S plot, individual metabolites and functional groups (X variables) can be pinpointed to be responsible for the bioactivity. This statistical model would assist in targeting the bioactive natural products for further isolation work [22]. Remarkably, SBT348 was the common outlier for both the HRMS and NMR data sets. Predicted antitrypanosomal active metabolites had molecular weights either from 100 to 200 Da for *Streptomyces* sp. SBT348 or 900 to 990 Da for *Nonomuraea* sp. SBT364 (Fig 5B). Strains of the genus *Nonomuraea* sp. were previously reported to produce glycopeptide antibiotics, anti-tumor cyclic tetrapeptides, and trehalose-derived metabolites [64–66], which were compatible to the dereplication results of the HRMS data. The active quadrant was also populated with mass ion peaks between 200 and 600 Da found in SBT348 as shown in 5B and further listed in S2 Table. Majority (12 out of 14) of these major compounds listed in S2 Table were aromatic as indicated by their RDBE (Ring Double Bond Equivalents) with values ≥4. On the other hand, the outlying chemical shifts obtained from the ^1^H NMR data (Fig 5D) corroborated with the compounds dereplicated from the HRMS data for SBT348, which included phenolic chemical shifts from 10 to 12 ppm for the O*H* along with their shielded *ortho* protons adjacent to the OH substituent observed at 6.0 to 6.9 ppm. Expansion of the lower left quadrant (Fig 5D) representing those of the active group revealed not only a higher distribution of chemical shifts typical to phenolic or aniline natural products but as well as resonances for α proton of amino acids from 4.01 to 5.55 and their corresponding amide protons with NMR shifts at 7.51 to 10 ppm for a peptide structure. According to the dereplication results, these chemical shifts were compatible with the peptide metabolites found in *Rhodococcus* sp. SBT367, the other outlier disclosed on the OPLS-DA scores plot of the ^1^H NMR data set (Fig 5C). *Rhodococcus* strains have also been described in the literature to yield peptides [67,68]. From the Milos Collection, SBT348 was therefore prioritized for scale-up and further isolation work based on the results of the dereplication study, PCA, and OPLS-DA along with its antitrypanosomal activity.

#### Crete Collection

**Fig 6:**
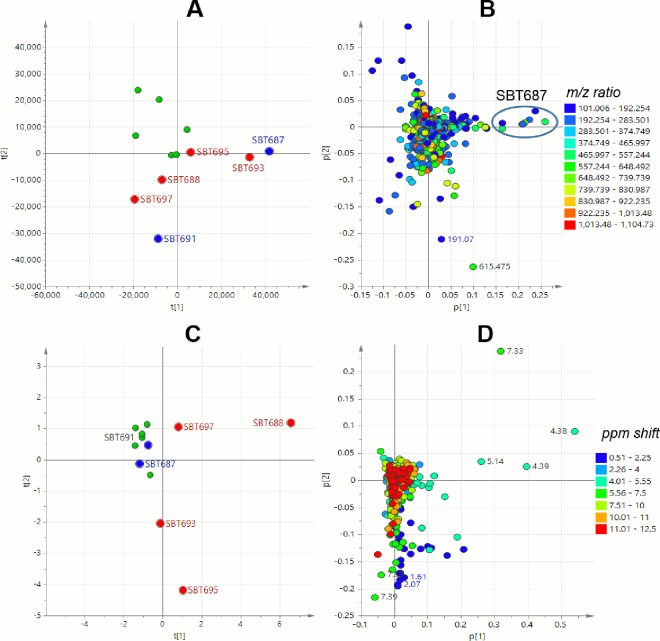
PCA analysis of 12 extracts from Crete collection. Scores plot (A) and loadings plot (B) of LC-HRMS data; scores plot (C) and loadings plot (D) of ^1^H NMR data of 12 extracts. For the scores plot (A and C) the outmost PCA outliers for the HRMS data were colored blue while those for the ^1^H NMR data were colored red.

**Fig 7:**
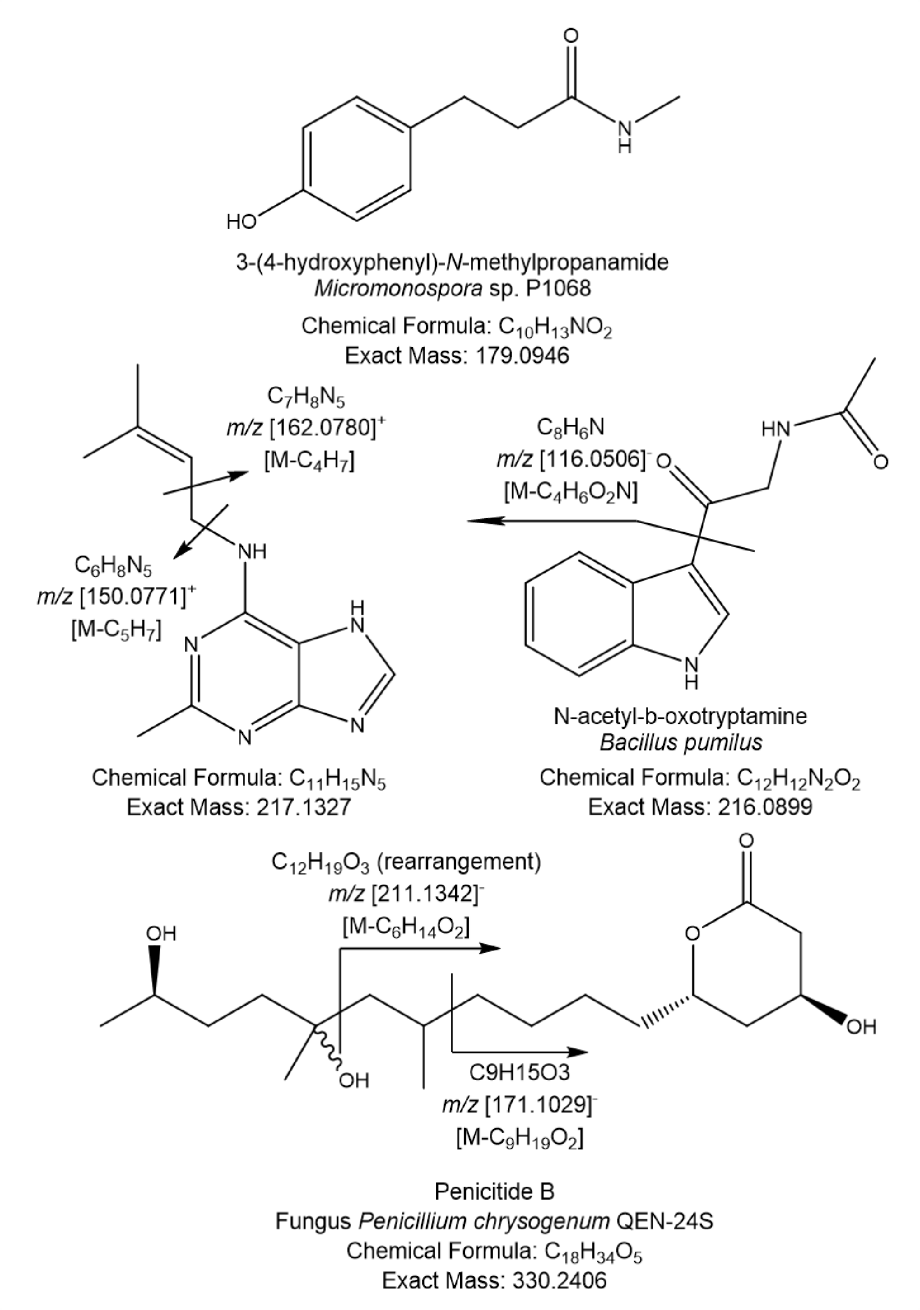
Dereplicated metabolites for the outlying mass ion peaks for *Micromonospora* sp. SBT687.

The 12 Crete isolates were also subjected to PCA analysis using their LC-MS and NMR data (Fig 6). We performed metabolomics analysis separately in order to avoid instrument errors (large shifting of *m/z* values) from different running batches by LC-MS. Using a two component model, PCA analysis of both the NMR and LC-MS data gave R2 values of 0.564 and 0.538, respectively. In the LC-HRMS PCA scores plot, *Streptomyces* sp. SBT691, *Micromonospora* species SBT687 and SBT693 were the outliers (Fig 6A). Only SBT687 was found active against *T*. *b*. *brucei* strain TC 221 (Table 1). From the loadings plot (Fig 6B), the outlying mass ion peaks for SBT687 were revealed at *m/z* 178.087 [M−H]^−^ for C_10_H_13_NO_2_, 218.14 [M+H]^+^ for C_11_H_15_N_5_, 274.131 [M−H]^−^ for C_13_H_17_N_5_O_2_, and 276.146 [M+H]^+^ for C_13_H_17_N_5_O_2_ or C_12_H_21_NO_6_. All these enumerated mass ion peaks gave no hits from any of the databases used, except for the mass ion peak at *m/z* 178.087 [M−H]^−^, which was putatively identified as 3-(4-hydroxyphenyl)-N-methylpropanamide, also earlier isolated from a *Micromonospora* species [69]. An extended list of major secondary metabolites for SBT 687 and their mass fragments were presented in S3 Table. SBT687 yielded a set of secondary metabolites with molecular weights ranging from 250 to 550 Da which are empirically chemically interesting (S3 Table, Fig 6B). The RDBEs of the metabolites were from 1 to 10, which was quite a wide range indicating that *Micromonospora* sp. SBT687 was producing a very heterogeneous set of secondary metabolites. The MS^2^ data along with the RDBE suggested the presence of aromatic compounds mostly between 200 and 300 Da (peak ID nos. 1, 6, 7, 10, and 12 in S3 Table) and adenine analogues (peak ID nos. 9 and 11 in S3 Table, Fig 7); as well as aliphatic type of compounds from 250 to 550 Da which included a sulfated compound (peak ID no. 2 in S3 Table), peptides (peak ID nos. 4, 13 and 18 in S3 Table); and hydroxylated lipids, lactone, or polyketides (peak ID nos. 3, 15, 16, and 17 in S3 Table). The mass ion peaks at *m/z* 215.083 [M−H]^−^ and 329.234 [M−H]^−^ gave hits from the AntiMarin Database 2013. The mass ion peak at *m/z* 215.083 [M−H]^−^ was tentatively identified as N-acetyl-β-oxotryptamine as further implicated by the MS^2^ fragment ion at *m/z* 116.0506 (Fig 7) that is characteristic of an indole moiety. This compound was previously isolated from marine bacterium *Bacillus pumilus* and exhibited inhibition activity against the growth of *Trypanosoma cruzi* with IC_50_ value of 19.4 μM [70]. While the mass ion peak at *m/z* 329.234 [M−H]^−^ was likely identified as penicitide B, (*Z*)-9, 10, 11-trihydroxyoctadec-12-enoic acid, and (9*R*, 10*R*, *E*)-6, 9, 10-trihydroxyoctadec-7-enoic acid, all of which are fungal metabolites. However, fragments ions observed at *m/z* 211.134 and 171.103 were compatible to peniticide B in which the molecule underwent a rearrangement by losing two protons (Fig 7) Peniticide B was previously described from the endophytic fungus *Penicillium chrysogenum* QEN-24S isolated from an algae of genus *Laurencia*, but there have been no report of its biological activities [71].

*Streptomyces* sp. SBT688 and *Micromonospora* sp. SBT695 were identified as the outliers in the PCA analysis of the ^1^H NMR data of the 12 Crete samples (Fig 6C). The unique chemical shifts of these two outliers (Fig 6D) were flaunted by aromatic (7.5 to 9.0 ppm) and heteroatom bound aliphatic protons (4.5 to 5.0 ppm). The antitrypanosomally active extract of *Micromonospora* sp. SBT687 was situated in the middle of the scores plot with higher density of exchangeable aromatic protons (10.0 to 12.5 ppm) shown by the loadings plot such as those of the indolic N*H* found in N-acetyl-β-oxotryptamine and the phenolic O*H* in 3-(4-hydroxyphenyl)-N-methylpropanamide.

Similar to *Streptomyces* sp. SBT348, it seems that the presence of exchangeable aromatic protons plays an important role on the antitrypanosomal activity of SBT687 extract. SBT687 was chosen for further scale-up and isolation work due to the diverse chemistry of its secondary metabolites which did not give any hits from the available databases used in this study. The diverse chemistry of the natural products associated with *Micromonospora* sp. SBT687 was revealed by the COSY spectrum of the crude extract (S5 Fig) which can be corroborated with the dereplication result obtained from the HRMS data.

As shown in this study, multivariate analysis by PCA was necessary to illustrate the occurrence of metabolites unique to the outlier strains which could conveniently corroborate the dereplication results. The PCA loading plots pin points the target metabolites that need to be validated by fragmentation and their NMR chemical shifts at which the dereplicated “hits” were further confirmed. From the dereplication study of complex extract mixtures, there are usually more than 1000 feature metabolites to be sorted out and with multivariate analysis plots, distinct features can define the uniqueness of a strain. This set of information was used to assist strain prioritization for future scale-up and isolation work of the new bioactive metabolite(s). The bioactive metabolites were further delineated using a supervised method of multivariate statistical analysis. This type of statistical method delimits the type of chemical scaffolds most responsible for the bioactivity. Such approach has been widely used as shown by earlier publications and references therein [22,24,26,72,73]. With regards to the NMR data, PCA analysis was a convenient way in removing the complex background peaks that belongs to the media and consequently defining the functional groups unique to the target strain as well as with that of the bioactive metabolite as we have shown in a previous paper [22,24].

## Conclusions

Out of 12 different sponge species collected from Greece in the current study we recovered 64 actinomycetes representing 23 different genera and including four putatively new species which were compared to their closest strains, including type strains. To our knowledge, the genera *Geodermatophilus*, *Microlunatus*, *Actinomycetospora*, *Modestobacter*, and *Promicromonospora* were isolated from marine sponges for the first time. Among these 64 isolates, eight organic extracts displayed activity against *Trypanosma brucei brucei* TC221. Two isolates *Streptomyces* sp. SBT348 (Milos collection) and *Micromonospora* sp. SBT687 (Crete collection) showed anti-trypanosomal activities as well as uniqueness in metabolomic profile and richness of unidentified natural products. These results prioritized SBT348 and SBT687 for scale-up, bioassay-guided fractionation, and isolation procedure. Our study demonstrated that utilizing metabolomics tools for screening and dereplication was an efficient approach to prioritize microorganisms from strain collections for drug discovery. The results also highlight marine sponges as rich source of new and bioactive actinomycetes as well as the importance of using new fermentation approaches to access novel actinomycete diversity.

## Supporting Information

S1 Fig Isolation of actinomycetes from different sponge species. (DOCX)

S2 Fig Maximum-likelihood tree of Micromonospora (A) and Streptomyces (B) isolates from the Crete (SBT6xx) and Milos collection (SBT3xx) as well as their nearest representative strains based on the 16S rRNA gene sequence. The trees are rooted at *Escherichia coli* strain KCTC 2441^T^ which serves as outgroup (not shown). All bootstrap values >50 (500 resamples) are given in percent at the nodes of the tree.
(DOCX)

S3 Fig ^1^H NMR (A), COSY (B) and HMBC (C) of ethyl acetate extract of bacterial isolate SBT349 showing anthranilic acid as the major component. (DOCX)

S4 Fig COSY spectrum of ethyl acetate extract of bacterial isolate SBT348. (DOCX)

S5 Fig COSY spectrum of ethyl acetate extract of bacterial isolate SBT687. (DOCX)

S1 Table Bacterial isolates from the Milos collection (2013) and Crete collection (2013). (DOCX)

S2 Table Selected major metabolites found in positive and negative ionization modes in Streptomyces SBT348. (P = positive mode; N = negative mode).
(DOCX)

S3 Table Selected major metabolites found in positive and negative ionization modes in *Micromonospora* SBT687. (P = positive mode; N = negative mode).
(DOCX)
