# Supplementary figures and images for: Biodiversity, Anti-Trypanosomal Activity Screening, and Metabolomic Profiling of Actinomycetes Isolated from Mediterranean Sponges
Source: PLoS One. 2015 Sep 25;10(9):e0138528. doi: 10.1371/journal.pone.0138528 (PMC4583450; doi:10.1371/journal.pone.0138528)

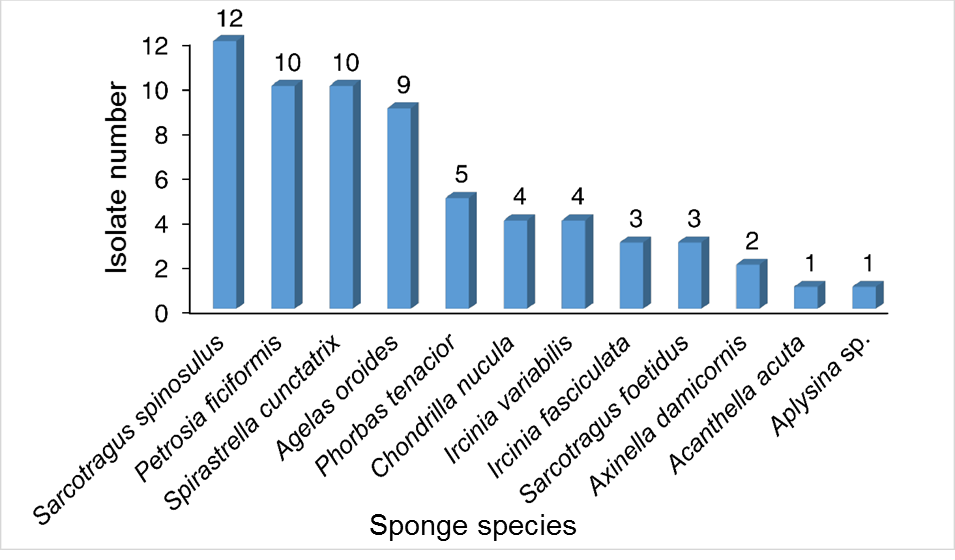


**S1 Fig. Isolation of actinomycetes from different sponge species.**

Supplement: S1 Fig — (DOCX) [file pone.0138528.s001.docx]
