# Supplementary material for: Biodiversity, Anti-Trypanosomal Activity Screening, and Metabolomic Profiling of Actinomycetes Isolated from Mediterranean Sponges
Source: PLoS One. 2015 Sep 25;10(9):e0138528. doi: 10.1371/journal.pone.0138528 (PMC4583450; doi:10.1371/journal.pone.0138528)
Supplement: S2 Fig — The trees are rooted at Escherichia coli strain KCTC 2441T which serves as outgroup (not shown). All bootstrap values >50 (500 resamples) are given in percent at the nodes of the tree. (DOCX) [file pone.0138528.s002.docx]

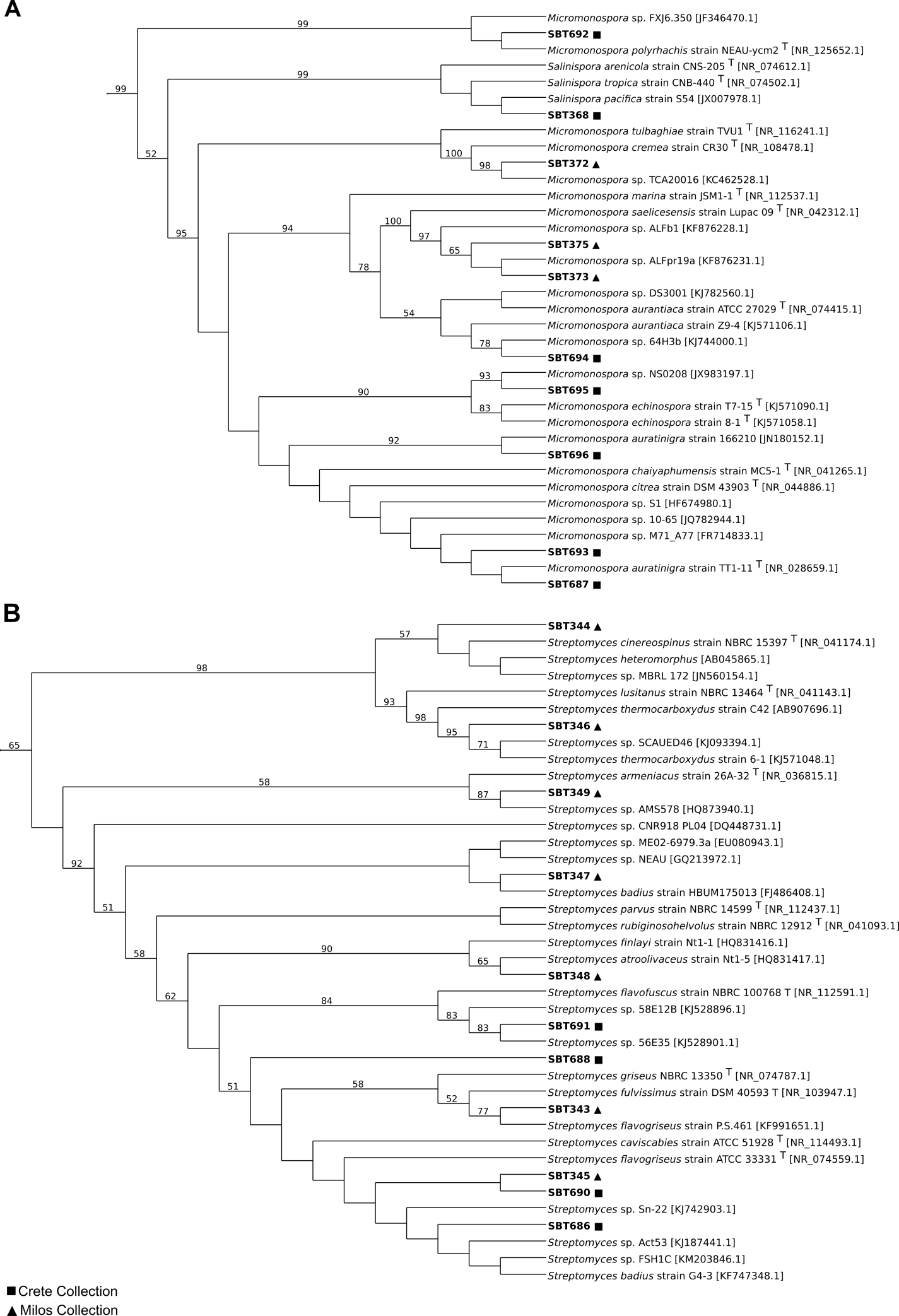
 **S2 Fig. Maximum-likelihood tree of Micromonospora (A) and Streptomyces (B) isolates from the Crete (SBT6xx) and Milos collection (SBT3xx) as well as their nearest representative strains based on the 16S rRNA gene sequence.** The trees are rooted at *Escherichia* *coli* strain KCTC 2441^T^ which serves as outgroup (not shown). All bootstrap values >50 (500 resamples) are given in percent at the nodes of the tree*.*
