# Supplementary material for: Biodiversity, Anti-Trypanosomal Activity Screening, and Metabolomic Profiling of Actinomycetes Isolated from Mediterranean Sponges
Source: PLoS One. 2015 Sep 25;10(9):e0138528. doi: 10.1371/journal.pone.0138528 (PMC4583450; doi:10.1371/journal.pone.0138528)
Supplement: S3 Fig — (DOCX) [file pone.0138528.s003.docx]

| A | B |
| --- | --- |
|  |  |
| C |  |
|  |  |

**S3 Fig.** **^1^H NMR (A), COSY (B) and HMBC (C) of** **ethyl acetate extract of bacterial isolate SBT349 showing anthranilic acid as the major component**
