# Supplementary material for: Biodiversity, Anti-Trypanosomal Activity Screening, and Metabolomic Profiling of Actinomycetes Isolated from Mediterranean Sponges
Source: PLoS One. 2015 Sep 25;10(9):e0138528. doi: 10.1371/journal.pone.0138528 (PMC4583450; doi:10.1371/journal.pone.0138528)
Supplement: S5 Fig — (DOCX) [file pone.0138528.s005.docx]

**S5 Fig. COSY spectrum of ethyl acetate extract of bacterial isolate SBT687**
