# Supplementary material for: Biodiversity, Anti-Trypanosomal Activity Screening, and Metabolomic Profiling of Actinomycetes Isolated from Mediterranean Sponges
Source: PLoS One. 2015 Sep 25;10(9):e0138528. doi: 10.1371/journal.pone.0138528 (PMC4583450; doi:10.1371/journal.pone.0138528)
Supplement: S1 Table — (DOCX) [file pone.0138528.s006.docx]

**S1 Table. Bacterial isolates from the Milos collection (2013) and Crete collection (2013)**

| Isolate Code | Sponge Source | Sequence Length | Closest Relative Strains by BLAST | % Sequence Identity |
| --- | --- | --- | --- | --- |
| SBT343 | *Petrosia ficiformis* | 1448 | *Streptomyces flavogriseus* strain P.S.461 | 99.65 |
| SBT344 | *Petrosia ficiformis* | 1450 | *Streptomyces cinereospinus* strain NBRC 15397 | 99.31 |
| SBT345 | *Agelas oroides* | 1398 | *Streptomyces* sp. 56E35 | 99.86 |
| SBT346 | *Petrosia ficiformis* | 1348 | *Streptomyces thermocarboxydus* | 99.93 |
| SBT347 | *Spirastrella cunctatrix* | 1178 | *Streptomyces* sp. CNR918 PL04 | 99.66 |
| SBT348 | *Petrosia ficiformis* | 1152 | *Streptomyces atroolivaceus* strain Nt1-5 | 99.91 |
| SBT349 | *Sarcotragus spinosulus* | 1339 | *Streptomyces* sp. AMS578 | 98.95 |
| SBT350 | *Chondrilla nucula* | 1262 | *Geodermatophilus obscurus* strain DSM 43160 | 98.41 |
| SBT351 | *Ircinia variabilis* | 1257 | *Agrococcus jenensis* strain B16 | 98.89 |
| SBT353 | *Sarcotragus spinosulus* | 1105 | *Dietzia maris* strain RB 30 | 99.55 |
| SBT354 | *Sarcotragus spinosulus* | 1186 | *Dietzia maris* strain RB 30 | 99.66 |
| SBT355 | *Sarcotragus spinosulus* | 1245 | *Dietzia maris* strain RB 30 | 100 |
| SBT356 | *Petrosia ficiformis* | 1358 | *Kocuria* sp. Drb9 | 99.41 |
| SBT357 | *Ircinia variabilis* | 1271 | *Kocuria rhizophila* strain XFB-BG | 100 |
| SBT358 | *Spirastrella cunctatrix* | 1265 | *Arthrobacter* sp. MR-17 | 99.92 |
| SBT359 | *Petrosia ficiformis* | 1231 | *Arthrobacter* sp. Als3 | 99.84 |
| SBT360 | *Agelas oroides* | 1253 | *Arthrobacter* sp. 19503 | 99.68 |
| SBT361 | *Sarcotragus spinosulus* | 1233 | *Gordonia terrae* strain 5-Sj-4-3-2-M | 99.92 |
| SBT362 | *Spirastrella cunctatrix* | 1312 | *Modestobacter roseus* strain KLBMP 1279 | 99.39 |
| SBT363 | *Sarcotragus foetidus* | 1351 | *Modestobacter multiseptatus* strain AA826 | 99.26 |
| SBT364 | *Sarcotragus foetidus* | 1336 | *Nonomuraea* sp. DLS-53 | 98.74 |
| SBT365 | *Spirastrella cunctatrix* | 1281 | *Microlunatus soli* strain CC-012602 | 97.97 |
| SBT366 | *Chondrilla nucula* | 1185 | *Nocardiopsis* sp. HB370 | 99.58 |
| SBT367 | *Spirastrella cunctatrix* | 1373 | *Rhodococcus opacus* strain S106 | 98.4 |
| SBT368 | *Spirastrella cunctatrix* | 1082 | *Salinispora pacifica* strain S54 | 99.72 |
| SBT370 | *Spirastrella cunctatrix* | 1205 | *Cellulosimicrobium* sp. 20.1 KSS | 99.92 |
| SBT371 | *Sarcotragus spinosulus* | 1170 | *Kytococcus* sp. HS24 | 99.74 |
| SBT372 | *Agelas oroides* | 1264 | *Micromonospora* sp. TCA20016 | 99.37 |
| SBT373 | *Chondrilla nucula* | 1193 | *Micromonospora* sp. ALFpr19a | 99.75 |
| SBT374 | *Petrosia ficiformis* | 1234 | *Actinomycetospora atypica* strain NEAU-st4 | 98.46 |
| SBT375 | *Sarcotragus spinosulus* | 1251 | *Micromonospora* sp. ALFpr19a | 99.84 |
| SBT376 | *Sarcotragus spinosulus* | 1184 | *Brachybacterium* sp. YB056 | 99.92 |
| SBT380 | *Sarcotragus spinosulus* | 1306 | *Knoellia* sp. S44CA | 99.92 |
| SBT381 | *Ircinia variabilis* | 1267 | Uncultured *Geodermatophilus* sp. clone BC100 | 99.84 |
| Isolate Code | **Sponge Source** | **Sequence Length** | **Closest Relative by BLAST** | **% Sequence Identity** |
| SBT686 | *Ircinia fasciculata* | 1287 | *Streptomyces* sp. Act53 | 99.77 |
| SBT687 | *Phorbas tenacior* | 1232 | *Micromonospora* sp. 10-65 | 99.59 |
| SBT688 | *Ircinia fasciculata* | 1329 | *Streptomyces badius* strain G4-3 | 99.85 |
| SBT689 | *Phorbas tenacior* | 1305 | *Nocardia araoensis* strain S107 | 99.54 |
| SBT690 | *Ircinia fasciculata* | 1260 | *Streptomyces* sp. Sn-22 | 99.92 |
| SBT691 | *Phorbas tenacior* | 1237 | *Streptomyces* sp. Sn-22 | 100 |
| SBT692 | *Phorbas tenacior* | 1084 | *Micromonospora* sp. FXJ6.350 | 99.82 |
| SBT693 | *Phorbas tenacior* | 1113 | *Micromonospora* sp. S1 | 99.73 |
| SBT694 | *Axinella damicornis* | 1262 | *Micromonospora* sp. DS3001 | 99.37 |
| SBT695 | *Agelas oroides* | 1292 | *Micromonospora echinospora* strain T7-15 | 99.37 |
| SBT696 | *Axinella damicornis* | 1200 | *Micromonospora auratinigra* strain 166210 | 99.92 |
| SBT697 | *Agelas oroides* | 1309 | *Promicromonospora umidemergens* strain 126185 | 99.62 |
