# Supplementary material for: Biodiversity, Anti-Trypanosomal Activity Screening, and Metabolomic Profiling of Actinomycetes Isolated from Mediterranean Sponges
Source: PLoS One. 2015 Sep 25;10(9):e0138528. doi: 10.1371/journal.pone.0138528 (PMC4583450; doi:10.1371/journal.pone.0138528)
Supplement: S2 Table — (P = positive mode; N = negative mode). (DOCX) [file pone.0138528.s007.docx]

| **Peak ID** | **ESI Mode** | **Molcular ion MS (m/z)** | **Rt (min)** | **Molecular Formula** | **MW** | **RDB** | **Hits** | **Fragment ion MS^2^ (m/z)** | **Fragment Formula**  **(+/-)** | **RDBE** | **Fragment ion MS^3^ (m/z)** | **Fragment Formula**  **(+/-)** | **RDBE** |
| --- | --- | --- | --- | --- | --- | --- | --- | --- | --- | --- | --- | --- | --- |
| 1 | N | 289.0683 | 1.5 | C_10_H_14_O_8_N_2_ | 290.0756 | 5 | No hits | 243.0626 | C_9_H_11_O_6_N_2_ | 5 | 200.0566 | C_8_H_10_O_5_N | 4 |
| 2 | N | 251.0565 | 3.8 | C_12_H_12_O_6_ | 252.0638 | 7 | No hits | 191.0353 | C_10_H_7_O_4_ | 7 | 147.0454 | C_9_H_7_O_2_ | 6 |
| 3 | P | 242.1023 | 4.0 | C_11_H_15_O_5_N | 241.095 | 5 | No hits | 225.0750 | C_11_H_14_O_5_ | 6 | 163.0750 | C_10_H_11_O_2_ | 6 |
|  |  |  |  |  |  |  |  | 207.0649 | C_11_H_12_O_4_ | 7 | 133.0646 | C_9_H_9_O | 6 |
| 4 | N | 241.1198 | 4.2 | C_11_H_18_O_4_N_2_ | 242.1271 | 4 | No hits | 197.1298 | C_10_H_17_O_2_N_2_ | 3 |  |  |  |
|  |  |  |  |  |  |  |  | 181.0985 | C_9_H_13_O_2_N_2_ | 4 |  |  |  |
|  |  |  |  |  |  |  |  | 154.1239 | C_9_H_16_ON | 2 |  |  |  |
| 5 | P | 219.1129 | 5.4 | C_12_H_14_O_2_N_2_ | 218.1056 | 5 | No hits | 191.1177 | C_11_H_16_ON_2_ | 6 | 159.0918 | C_10_H_11_N_2_ | 7 |
|  |  |  |  |  |  |  |  | 174.0914 | C_11_H_13_ON | 7 | 130.0653 | C_9_H_8_N | 7 |
|  |  |  |  |  |  |  |  | 120.0809 | C_8_H_11_N | 5 |  |  |  |
| 6 | N | 319.1766 | 7.0 | C_15_H_28_O_7_ | 320.1839 | 2 | No hits | 125.0973 | C_8_H_13_O | 2 | 97.06588 | C_6_H_9_O | 2 |
| 7 | N | 347.2079 | 10.3 | C_19_H_28_O_4_N_2_ | 348.2152 | 7 | No hits | 311.2233 | C_18_H_31_O_4_ | 3 |  |  |  |
| 8 | P | 271.0963 | 11.1 | C_12_H_15_O_6_N | 270.0890 | 6 | No hits | 252.0862 | C_12_H_15_O_5_N | 7 |  |  |  |
|  |  |  |  |  |  |  |  | 126.0549 | C_6_H_9_O_2_N | 4 |  |  |  |
|  |  |  |  |  |  |  |  | 108.0444 | C_6_H_7_ON | 5 |  |  |  |
| 9 | P | 325.2275 | 11.3 | C_21_H_28_ON_2_ | 324.2202 | 9 | No hits | 233.1639 | C_14_H_22_ON_2_ | 6 | 160.1117 | C_11_H_14_N | 6 |
|  |  |  |  |  |  |  |  |  |  |  | 148.1118 | C_10_H_14_N | 5 |
|  |  |  |  |  |  |  |  |  |  |  | 84.0807 | C_5_H_20_N | 2 |
| 10 | N | 206.0826 | 11.7 | C_11_H_13_O_3_N | 207.0899 | 6 | No hits | 164.0718 | C_9_H_10_O_2_N | 5 |  |  |  |
|  |  |  |  |  |  |  |  | 147.0454 | C_9_H_7_O_2_ | 6 |  |  |  |
| 11 | N | 399.2758 | 12.4 | C_22_H_40_O_6_ | 400.2831 | 3 | No hits | 313.2017 | C_17_H_29_O_5_ | 3 |  |  |  |
| 12 | P | 423.2717 | 12.4 | C_25_H_34_O_2_N_4_ | 422.2644 | 10 | No hits | 406.2481 | C_25_H_32_O_2_N_3_ | 12 |  |  |  |
| 13 | P | 250.1437 | 14.3 | C_14_H_19_O_3_N | 249.1365 | 6 | No hits | 232.1327 | C_14_H_19_O_2_N | 7 |  |  |  |
|  |  |  |  |  |  |  |  | 204.1379 | C_13_H_19_ON | 6 |  |  |  |
|  |  |  |  |  |  |  |  | 166.0858 | C_9_H_13_O_2_N | 5 |  |  |  |
|  |  |  |  |  |  |  |  | 120.0807 | C_8_H_11_N | 5 |  |  |  |
| 14 | N | 583.3497 | 15.2 | C_29_H_44_O_5_N_8_ | 584.357 | 12 | No hits | 329.1664 | C_22_H_21_ON_2_ | 13 |  |  |  |

**S2 Table. Selected major metabolites found in positive and negative ionization modes in Streptomyces SBT348.** (P = positive mode; N = negative mode)
