# Supplementary material for: Biodiversity, Anti-Trypanosomal Activity Screening, and Metabolomic Profiling of Actinomycetes Isolated from Mediterranean Sponges
Source: PLoS One. 2015 Sep 25;10(9):e0138528. doi: 10.1371/journal.pone.0138528 (PMC4583450; doi:10.1371/journal.pone.0138528)
Supplement: S3 Table — (P = positive mode; N = negative mode). (DOCX) [file pone.0138528.s008.docx]

| Peak ID  **S3 Table. Selected major metabolites found in positive and negative ionization modes in *Micromonospora* SBT687.** (P = positive mode; N = negative mode) | ESI Mode | Molcular ion MS (m/z) | Rt (min) | Molecular Formula | MW | RDB | Hits | Fragment ion MS^2^ (m/z) | Fragment Formula  (+/-) | RDBE | Fragment ion MS^3^ (m/z) | Fragment Formula  (+/-) | RDB |
| --- | --- | --- | --- | --- | --- | --- | --- | --- | --- | --- | --- | --- | --- |
| 1 | N | 279.0731 | 1.2 | C_11_H_12_O_5_N_4_ | 280.0804 | 8 | No hits | 135.0314 | C_5_H_2_ON_4_ | 6 |  |  |  |
|  |  |  |  |  |  |  |  | 143.0351 | C_6_H_7_O_4_ | 3 |  |  |  |
| 2 | P | 256.1754 | 2.4 | C_14_H_25_ONS | 255.1681 | 3 | No hits | 239.1482 | C_14_H_23_OS | 4 |  |  |  |
| 3 | P | 283.1751 | 3.0 | C_12_H_26_O_7_ | 282.1678 | 1 |  | 133.0857 | C_6_H_13_O_3_ | 1 |  |  |  |
|  |  |  |  |  |  |  |  | 177.1118 | C_8_H_17_O_4_ | 1 |  |  |  |
|  |  |  |  |  |  |  |  | 89.0597 | C_4_H_9_O_2_ | 1 |  |  |  |
|  |  |  |  |  |  |  |  | 239.1484 | C_10_H_23_O_6_ | 0 |  |  |  |
| 4 | P | 388.2543 | 4.1 | C_17_H_33_O_5_N_5_ | 387.2470 | 4 | No hits | 371.2278 | C_17_H_31_O_5_N_4_ | 5 | 353.2168 | C_17_H_29_O_4_N_4_ | 6 |
|  |  |  |  |  |  |  |  |  |  |  | 327.2011 | C_15_H_27_O_4_N_4_ | 5 |
|  |  |  |  |  |  |  |  |  |  |  | 309.1907 | C_15_H_25_O_3_N_4_ | 6 |
| 5 | P | 432.2805 | 4.5 | C_19_H_37_O_6_N_5_ | 431.2732 | 4 | No hits | 415.2527 | C_19_H_35_O_6_N_4_ | 4 | 397.2434 | C_19_H_33_O_5_N_4_ | 6 |
|  |  |  |  |  |  |  |  |  |  |  | 371.2277 | C_17_H_32_O_5_N_4_ | 5 |
|  |  |  |  |  |  |  |  |  |  |  | 327.2015 | C_15_H_27_O_4_N_4_ | 5 |
|  |  |  |  |  |  |  |  |  |  |  | 309.1908 | C_15_H_25_O_3_N_4_ | 6 |
|  |  |  |  |  |  |  |  |  |  |  | 283.1752 | C_13_H_23_O_3_N_4_ | 5 |
| 6 | P | 210.1124 | 4.6 | C_11_H_15_O_3_N | 209.1052 | 4 | No hits | 151.0751 | C_9_H_11_O_2_ | 5 | 119.0494 | C_8_H_7_O | 6 |
|  |  |  |  |  |  |  |  | 168.1015 | C_9_H_14_O_2_N | 5 | 95.0494 | C_6_H_7_O | 4 |
|  |  |  |  |  |  |  |  | 181.1333 | C_10_H_17_ON_2_ | 4 | 91.0545 | C_7_H_7_ | 5 |
| 7 | N | 224.0932 | 5.1 | C_11_H_15_O_4_N | 225.1004 | 5 | No hits | 178.0875 | C_10_H_12_O_2_N | 5 | 136.0768 | C_8_H_10_ON | 4 |
|  |  |  |  |  |  |  |  |  |  |  | 119.0523 | C_8_H_7_O | 5 |
| 8 | P | 520.3331 | 5.1 | C_23_H_45_O_8_N_5_ | 519.3258 | 4 | No hits | 503.3055 | C_23_H_43_O_8_N_4_ | 5 | 485.2954 | C_23_H_41_O_7_N_4_ | 6 |
|  |  |  |  |  |  |  |  |  |  |  | 459.2798 | C_21_H_39_O_7N4_ | 5 |
|  |  |  |  |  |  |  |  |  |  |  | 415.2534 | C_19_H_35_O_6_N_4_ | 5 |
|  |  |  |  |  |  |  |  |  |  |  | 397.2430 | C_19_H_33_O_5_N_4_ | 6 |
|  |  |  |  |  |  |  |  |  |  |  | 371.2277 | C_17_H_31_O_5_N_4_ | 5 |
|  |  |  |  |  |  |  |  |  |  |  | 353.2167 | C_17_H_29_O_4_N_4_ | 6 |
|  |  |  |  |  |  |  |  |  |  |  | 327.2013 | C_15_H_27_O_4_N_4_ | 5 |
|  |  |  |  |  |  |  |  |  |  |  | 309.1906 | C_15_H_25_O_3_N_4_ | 6 |
|  |  |  |  |  |  |  |  |  |  |  | 283.1751 | C_13_H_23_O_3_N_4_ | 5 |
|  |  |  |  |  |  |  |  |  |  |  | 177.1122 | C_8_H_17_O_4_ | 1 |
| 9 | P | 218.1399 | 5.7 | C_11_H_15_N_5_ | 217.1327 | 7 | No hits | 162.0770 | C_7_H_8_N_5_ | 7 |  |  |  |
|  |  |  |  |  |  |  |  | 150.0771 | C_6_H_8_N_5_ | 6 |  |  |  |
|  |  |  |  |  |  |  |  |  |  |  |  |  |  |
| 10 | N | 215.0829 | 6.5 | C_12_H_12_O_2_N_2_ | 216.0902 | 8 | N-acetyl-β-oxotryptamine | 116.0506 | C8H6N | 6 |  |  |  |
| 11 | N | 274.1312 | 6.8 | C_13_H_17_O_2_N_5_ | 275.1385 | 8 | No hits | 217.1098 | C_11_H_13_ON_4_ | 7 | 202.0864 | C_12_H_12_O_2_N | 7 |
| 12 | P | 284.1393 | 6.8 | C_16_H_17_O_2_N_3_ | 283.1320 | 10 | No hits | 267.1122 | C_16_H_15_O_2_N_2_ | 11 |  |  |  |
|  |  |  |  |  |  |  |  | 170.0597 | C_11_H_8_ON | 8 |  |  |  |
|  |  |  |  |  |  |  |  | 130.0650 | C_9_H_8_N | 7 |  |  |  |
|  |  |  |  |  |  |  |  | 132.0806 | C_9_H_10_N | 6 |  |  |  |
| 13 | P | 227.1753 | 8.2 | C_12_H_22_O_2_N_2_ | 226.1681 | 3 | No hits | 199.1800 | C_11_H_23_ON_2_ | 2 | 154.1587 | C_10_H_20_N | 2 |
|  |  |  |  |  |  |  |  | 182.1536 | C_11_H_20_ON | 3 | 86.0964 | C_5_H_12_N | 1 |
| 14 | N | 329.2340 | 12.0 | C_18_H_34_O_5_ | 330.2413 | 2 | penicitide B | 229.1448 | C_12_H_21_O_4_ | 2 |  |  |  |
|  |  |  |  |  |  |  |  | 211.1342 | C_12_H_19_O_3_ | 3 |  |  |  |
|  |  |  |  |  |  |  |  | 171.1029 | C_9_H_15_O_3_ | 2 |  |  |  |
| 15 | N | 525.3806 | 13.4 | C_30_H_54_O_7_ | 526.3879 | 4 | No hits | 311.2589 | C_19_H_35_O_3_ | 2 | 293.2490 | C_19_H_33_O_2_ | 3 |
| 16 | N | 523.3650 | 15.0 | C_30_H_52_O_7_ | 524.3722 | 5 | No hits | 309.2434 | C_19_H_33_O_3_ | 3 | 291.2332 | C_19_H_31_O_2_ | 4 |
|  |  |  |  |  |  |  |  |  |  |  | 233.1914 | C_16_H_25_O | 4 |
| 17 | P | 509.3840 | 17.0 | C_30_H_52_O_6_ | 508.3767 | 5 | No hits | 491.3731 | C_30_H_51_O_5_ | 6 | 311.1847 | C_17_H_27_O_5_ | 5 |
|  |  |  |  |  |  |  |  | 473.3626 | C_30_H_49_O_4_ | 7 | 293.1749 | C_17_H_25_O_4_ | 6 |
|  |  |  |  |  |  |  |  | 455.3521 | C_30_H_47_O_3_ | 8 | 259.2419 | C_19_H_31_ | 5 |
|  |  |  |  |  |  |  |  | 437.3415 | C_30_H_45_O_2_ | 9 |  |  |  |
|  |  |  |  |  |  |  |  | 295.2631 | C_19_H_35_O_2_ | 3 |  |  |  |
|  |  |  |  |  |  |  |  | 277.2526 | C_19_H_33_O | 4 |  |  |  |
|  | N | 507.3686 | 17.0 | C_30_H_52_O_6_ | 508.3767 | 5 | No hits | 461.3639 | C_29_H_49_O_4_ | 5 |  |  |  |
| 18 | N | 553.3755 | 17.1 | C_27_H_50_O_6_N_6_ | 554.3828 | 6 | No hits | 405.3002 | C_23_H_39_O_3_N_3_ | 6 | 311.2592 | C_19_H_35_O_3_ | 2 |
| 19 | P | 530.3547 | 23.3 | C_25_H_47_O_7_N_5_ | 529.3474 | 5 | No hits | 513.3259 | C_25_H_45_O_7_N_4_ | 6 | 495.3162 | C_25_H_43_O_6_N_4_ | 7 |
|  |  |  |  |  |  |  |  |  |  |  | 371.2273 | C_17_H_31_O_5_N_4_ | 5 |
|  |  |  |  |  |  |  |  |  |  |  | 187.1328 | C_10_H_19_O_3_ | 2 |
